# An overview of bioinformatics courses delivered at the academic level in Italy: Reflections and recommendations from BITS

**DOI:** 10.1371/journal.pcbi.1010846

**Published:** 2023-02-13

**Authors:** Roberto Marangoni, Vitoantonio Bevilacqua, Mario Cannataro, Bruno Hay Mele, Giancarlo Mauri, Anna Marabotti, on behalf of the BITS Training&Teaching group

**Affiliations:** 1 Bioinformatics Italian Society (BITS), Roma, Italy; 2 Department of Biology, University of Pisa, Pisa, Italy; 3 Department of Electrical and Information Engineering, Polytechnic University of Bari, Bary, Italy; 4 Department of Medical and Surgical Sciences, University Magna Graecia of Catanzaro, Catanzaro, Italy; 5 Department of Biology, University of Naples “Federico II”, Naples, Italy; 6 Department of Informatics, Systems and Communication, University of Milano-Bicocca, Milan, Italy; 7 Department of Chemistry and Biology “A. Zambelli”, University of Salerno, Fisciano, Italy; SIB Swiss Institute of Bioinformatics, SWITZERLAND

## Abstract

In Italian universities, bioinformatics courses are increasingly being incorporated into different study paths. However, the content of bioinformatics courses is usually selected by the professor teaching the course, in the absence of national guidelines that identify the minimum indispensable knowledge in bioinformatics that undergraduate students from different scientific fields should achieve. The Training&Teaching group of the Bioinformatics Italian Society (BITS) proposed to university professors a survey aimed at portraying the current situation of bioinformatics courses within undergraduate curricula in Italy (i.e., bioinformatics courses activated within both bachelor’s and master’s degrees). Furthermore, the Training&Teaching group took a cue from the survey outcomes to develop recommendations for the design and the inclusion of bioinformatics courses in academic curricula. Here, we present the outcomes of the survey, as well as the BITS recommendations, with the hope that they may support BITS members in identifying learning outcomes and selecting content for their bioinformatics courses. As we share our effort with the broader international community involved in teaching bioinformatics at academic level, we seek feedback and thoughts on our proposal and hope to start a fruitful debate on the topic, including how to better fulfill the real bioinformatics knowledge needs of the research and the labor market at both the national and international level.

## Introduction

The publication of the first draft of the human genome in 2001 [1,2] represented a cornerstone for scientific knowledge and raised awareness of the fundamental role played by bioinformatics, without which this achievement would not have been possible [3]. In subsequent years, the enormous amount of biological data produced posed new challenges in the design and development of new databases and algorithms able to process the generated layers of knowledge. In turn, this has enabled a “virtuous circle” that enforced bioinformatics’ fundamental role in data management and processing in modern biology and medicine [4]. Besides, the increased need for algorithms and computational tools in biological research also prompted computer scientists to broaden their research horizons [5]. This raised an increasing need for equipping students with at least basic bioinformatics knowledge, skills, and abilities. This led several Italian universities in the last 2 decades to incorporate bioinformatics courses (for the explanation of the terminology, please refer to S1 Text) into various academic study paths belonging to different scientific areas, both at bachelor’s (BSc) and master’s (MSc) degree levels. The learning outcomes and content of these bioinformatics courses, however, are generally designed according only to the perceived needs of the local academic contexts and by the teacher’s prevailing scientific interest or skills.

In March 2021, in order to provide a snapshot of bioinformatics courses in Italian universities, the Italian Society of Bioinformatics (BITS) designed a survey asking BITS members involved in bioinformatics teaching at academic level to provide information about bioinformatics semester courses taught at their universities. The survey was not anonymous, to allow us to contact the compiler in case of need (no sensitive/personal information was included in the survey), and we decided to insert in the survey the following questions: the name of the course (as we were aware that often, courses with a bioinformatics content are not delivered with the simple name “Bioinformatics”); the code identifying, in Italy, the academic discipline (in Italian: “settore scientifico-disciplinare”, SSD) (for the explanation of the terminology, please refer to S1 Text) of the teacher and of the course, to see if the bioinformatics content was delivered by teachers and in courses properly classified in a SSD that includes bioinformatics as a topic; if the course is mandatory or optional in the study path of the student; the degree level (BSc or MSc) of the study path in which the course is delivered, and the broad scientific area of the study path (we didn’t want to dissect each scientific area precisely, but only wanted to know if these courses were placed in study paths belonging to a life science/medicine (“BIO”) or a computer science/engineering (“INFO”) scientific area). Moreover, we asked to report the number of European Credit Transfer and Accumulation System (ECTS) attributed to the course and the number of hours globally attributed to the course for classroom lessons and exercises (it is worth noting that in Italy, it is mandatory to attribute no less than 6 ECTS to a single course, unless it is a module of a more general course, or under exceptional circumstances to be documented by the relevant academic body). To understand what topics were most commonly covered in the bioinformatics courses, we selected the topics explicitly covered in the 2 most popular textbooks in Italy for teaching bioinformatics (as confirmed by the results of our survey), written by authors belonging to the Italian Bioinformatics Society [6,7]. The questions included in the survey are reported in Table 1.

**Table 1:** Questions included in the survey sent to the Bioinformatics Italian community.

Question	Format for the answer
Name of the course	Free text
Academic discipline of the teacher	Free text (code of the discipline: https://www.cun.it/uploads/storico/settori_scientifico_disciplinari_english.pdf)
Academic discipline in which the course is classified	Free text (code of the discipline: https://www.cun.it/uploads/storico/settori_scientifico_disciplinari_english.pdf)
Type of the course	2 options: Mandatory/optional
Degree level of the study path in which the course is delivered	2 options: BSc/MSc
Scientific area of the study path in which the course is delivered	Options: biology or biomedicine/computer science or engineering/other (to be specified)
University	Free text
ECTS assigned to the course	Free text
Total hours for the front lessons/classroom exercises	Free text
Topics of the course	Select all that apply among: • Fundamentals of computer science • Fundamentals of biology and genomics • Biological databases • Phylogenetic trees • Sequencing, NGS, assembly, reads elaboration • Transcriptomics, functional genomics • Proteomics and metabolomics • Structural bioinformatics • Molecular mechanics and molecular dynamics • Docking • Systems biology, metabolic networks • Algorithms for bioinformatics • Dynamics programming algorithms • Sequence alignments algorithms • Graphs • Clustering • Introduction to Machine Learning • Neural networks • Hidden Markov Models • Support Vector Machines • Introduction to Deep Learning • Basics of programming • Programming in Python/Perl/C or other languages • Use of R and specific packages • Bioconductor • Other (to be specified)
Suggested textbooks	Free text
Other comments	Free text

The original survey was in Italian.

The survey was delivered by means of an electronic platform (Google Modules) and made available for about 2 months; all members of the BITS (about 400 people as of the date of the survey, of whom, however, only a portion were in charge of teaching activities in bioinformatics at the university level) were informed of the survey via internal mailing lists, and solicited both to compile the survey and to spread the information to other colleagues. We obtained 51 answers on a voluntary basis (S1 Data), for 47 of which we were able to identify the geographical provenience (S1 Fig). To the best of our knowledge, this was the first and most extensive example in Italy of an effort to conduct a fact-finding survey related to the way bioinformatics is taught. The answers represent probably a little sample (we estimated between 10% to 30%) with respect to the whole academic scenario of bioinformatics teaching in Italy, therefore, we want to point out that we do not consider this sample as fully representative; however, the results are in line with our empirical knowledge of the Italian scenario of bioinformatics teaching. The results of the survey were analyzed and discussed during the 2021 Annual Meeting of the Bioinformatics Italian Society, held virtually because of the COVID-19 pandemic (July 1 and 2, http://bioinformatics.it/bits2021) and are summarized here.

**Fig 1:**
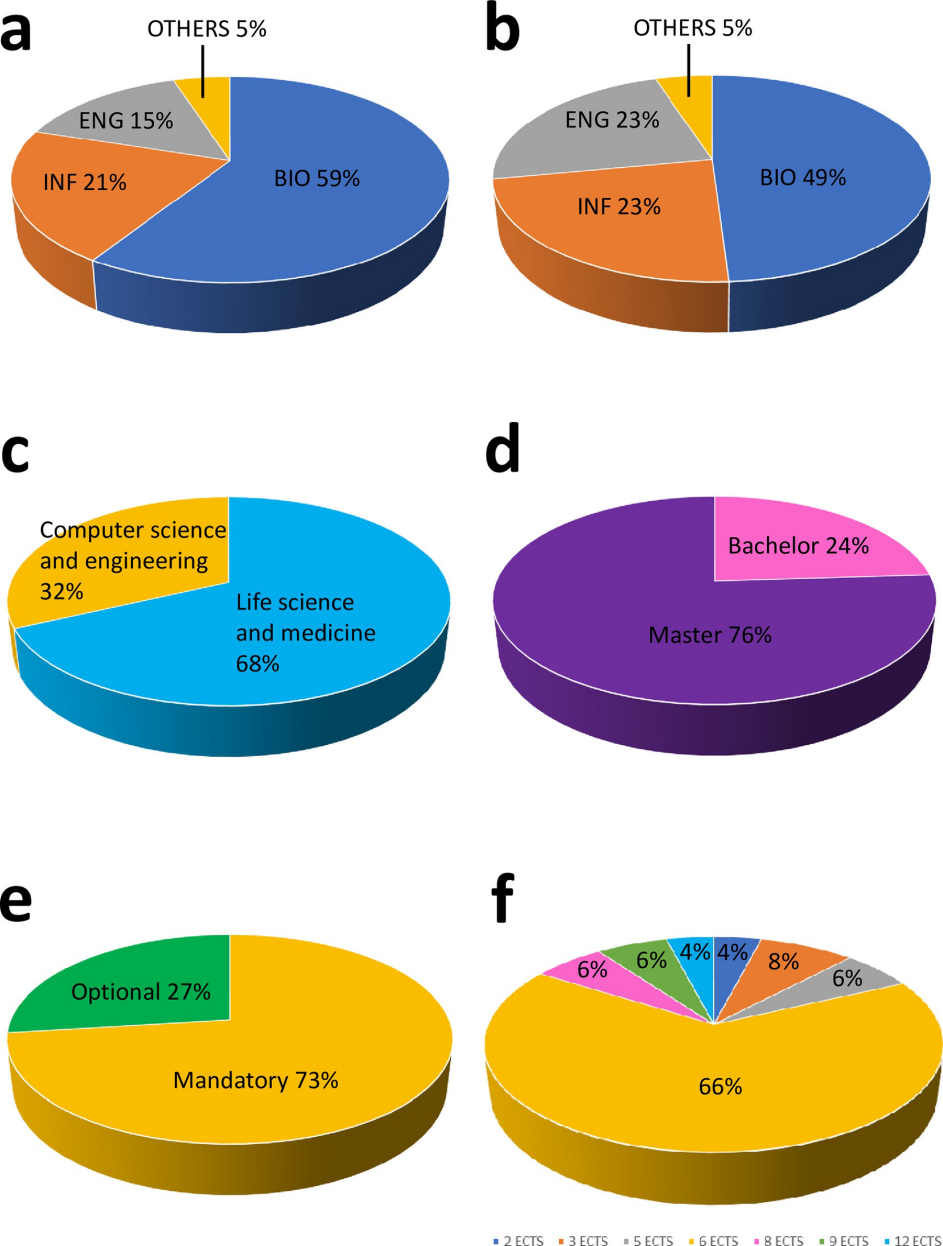
Pie charts describing the general results obtained from the survey. Panel (a): Academic disciplines of the teachers who responded to the survey. BIO: life science/medicine disciplines; INF: computer science disciplines; ENG: engineering disciplines; Others: other scientific disciplines. Panel (b): Academic disciplines attributed to the bioinformatics courses. Label meaning is the same as in panel (a). Panel (c): Scientific area to which the study path in which the bioinformatics course is delivered belongs. Panel (d): Degree level in which the bioinformatics course is delivered. Panel (e): Type of the course (either mandatory or optional). Panel (f): ECTS attributed to the bioinformatics courses reported in the survey.

It turned out that roughly, 60% of respondent teachers in Bioinformatics are from life science academic disciplines (mainly molecular biology, biochemistry, and genetics), 20% are from the computer science area, 15% are computer engineers, with a marginal presence of physicians or statisticians (Fig 1a; for details about the SSDs, please refer to S2A Fig). The SSD in which the bioinformatics course was classified belongs to the life science/medicine area in nearly half of the cases, indicating that for some courses, there is no correspondence between the academic discipline of the teacher and that of the course (Fig 1b; for details about the SSDs, please refer to S2B Fig). Bioinformatics courses are delivered more in study paths belonging to life science and medicine scientific area (68%) than in study paths belonging to the computer science/engineering scientific area (32%) (Fig 1c). Most of the bioinformatics courses (76%) are delivered in a study path belonging to an MSc (Fig 1d), and most of them are delivered as a mandatory course (Fig 1e). The number of ECTS attributed to bioinformatics courses in different study paths varies from 2 to 12 (Fig 1f), with a median of 6 ECTS. A slightly lower median ECTS is attributed to bioinformatics courses embedded in a BSc study path (5.6 ECTS) with respect to bioinformatics courses embedded in an MSc study path (6.3). The median ECTS attributed to bioinformatics courses delivered in a study path belonging to life science scientific area is slightly lower with respect to the median ECTS attributed to bioinformatics courses delivered in a study path belonging to computer science/engineering scientific area (6 and 6.87, respectively). On average, 53 hours are attributed to the bioinformatics courses, which means that approximately 1 in 6 ECTS is devoted to practical activities (in Italy, usually 1 ECTS corresponds to 8 hours of front lessons).

**Fig 2:**
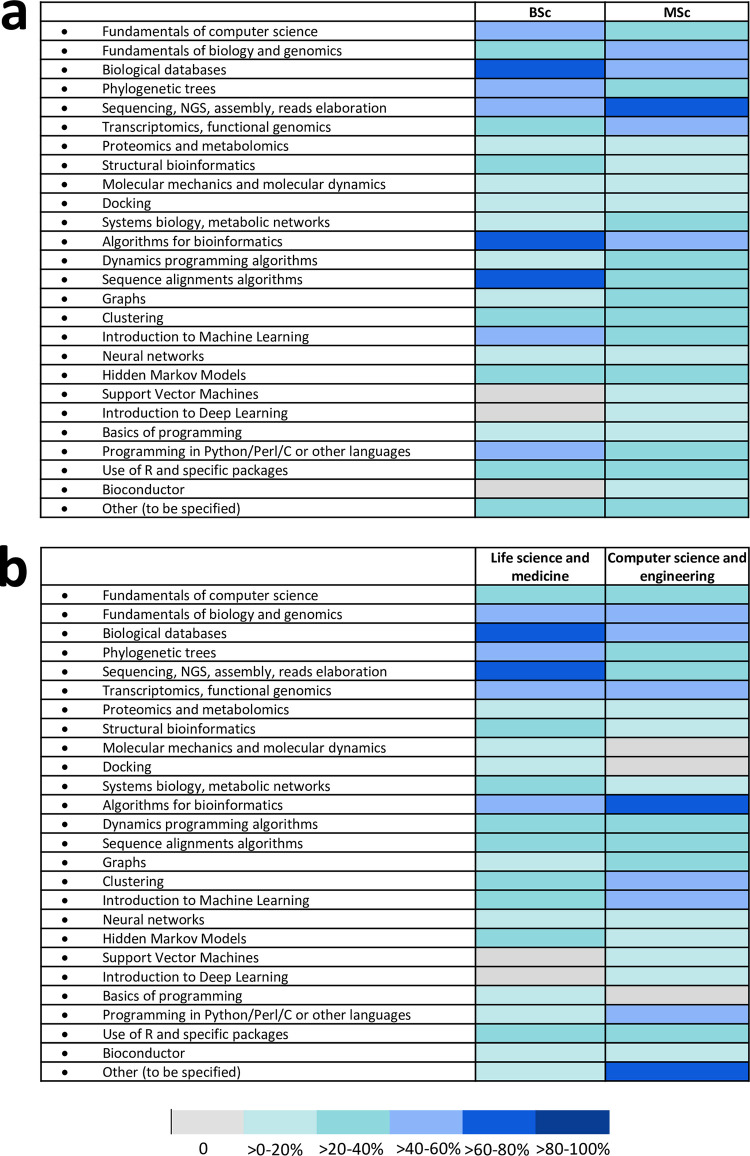
Heat map describing how the different topics are distributed in the bioinformatics courses delivered in different degrees levels (panel (a)) or in study paths belonging to different scientific areas (panel (b)). Different colors refer to the different percentages of occurrence of each topic in the bioinformatics courses.

The most frequently covered topics relate to databases of biological interest, alignment algorithms, genomics and transcriptomics, clustering, and the use of specific programs written in various programming languages such as R or Python; structural and applied bioinformatics in proteomics and metabolomics, applications of molecular mechanics, and bioinformatics applications of deep learning are generally less covered (S3 Fig). In our opinion, the choice of these topics is acceptable and associated with the increasing availability of data obtained from genomics and transcriptomics projects, with the consequent need to know how to consult and analyze them, while other applications seem to have a more niche character or are still perceived as too innovative to be included in courses for undergraduate students. It is possible to observe that bioinformatics courses delivered in a path study in BSc degrees tend to include more frequently the following topics: fundamentals of computer science, biological databases, phylogenetic trees, structural bioinformatics, algorithms for bioinformatics, dynamic programming algorithms, sequence alignment algorithms, introduction to machine learning, and programming in Python/Perl/C or other languages. On the contrary, topics included more frequently in bioinformatics courses delivered in a path study in MSc degrees are: fundamentals of biology and genomics, sequencing and related topics, transcriptomics and functional genomics, systems biology and metabolic networks, graph theory (Fig 2a). Biological databases, phylogenetic trees, sequencing and related topics, structural bioinformatics, systems biology and metabolic networks, and Hidden Markov Models are topics that tend to be more frequently treated in bioinformatics courses delivered in study paths belonging to life science and medicine scientific area, whereas algorithms for bioinformatics, graph theory, clustering analysis, introduction to Machine Learning, programming in Python/Perl/C or other languages tend to be more frequently treated in bioinformatics courses delivered in study paths belonging to computer science and engineering scientific areas. Moreover, in these 2 scientific areas, the courses tended to be more customized with topics other than those explicitly included in the survey, such as Matlab Toolbox Bioinformatics, gene expression data production, bio-ontologies, enrichment analysis, semantic similarity analysis, microarrays and mass spectrometry data, regular expressions, command line to manipulate data (Fig 2b). It seems therefore that the focus of bioinformatics courses in the different study paths is different: in life science and medicine, it seems to be more important to convey the biological information that can be obtained by the mining of the data, whereas in engineering and computer science, it seems to be more important to develop new strategies to analyze the data, irrespective of their biological origin.

Reflecting on the results of this survey, while acknowledging its limitations, and considering that bioinformatics approaches and technologies will become increasingly important in science in the future, BITS decided to start an effort to develop recommendations for teaching bioinformatics at the academic level. These recommendations were partially based on the survey results and their analysis, and partially based on the educational experience of the participants of the Training&Teaching group in the Italian academic scenario. In particular, the analysis of the ECTS and hours attributed to the bioinformatics courses and of the topics treated in the courses constituted the backbone of the suggestions about the recommended content to include in the course. Our intent was to suggest to the Italian teachers of bioinformatics courses what minimum indispensable knowledge in bioinformatics BITS believes is essential for a life scientist, or an engineer, or a computer scientist, and therefore should be present in a bioinformatics course embedded in a more general study path in life science/medicine, or engineering/computer science, both in BSc and MSc degrees. The Training&Teaching group of the Society focused on this work and concluded this activity in March 2022 by generating a document that includes recommended reference content for the different levels and scientific areas to which the degrees refer, and additional recommendations to frame the bioinformatics courses within a context that allows its optimal fruition by students. This document has been posted on the BITS website [8].

We decided to present its contents here, because we would like to share it with the international bioinformatics community and, hopefully, open a discussion on this topic that will allow us to improve our future directions.

### Recommended contents for the Bioinformatics courses

Considering that the placement of bioinformatics courses is prevalent in study paths belonging to 2 main areas (life science/medicine versus computer science/engineering), and considering the different backgrounds and goals of students in these 2 areas, BITS believes it is appropriate to indicate the different skill sets of students according to the scientific area of their degree programs. Consequently, different recommended contents have been outlined, referring to those bioinformatics courses to be placed in a BSc degree and those to be placed in an MSc degree for the 2 main scientific areas mentioned above. Our survey highlighted that the median ECTS currently attributed to bioinformatics courses is 6, of which 1 is spent for practical activities (guided exercises, hands-on, etc.). We felt that this teaching load is appropriate in the case of a course to be delivered in a BSc degree (180 ECTS), possibly increasing the time devoted to practicing to 2 ECTS. In the case of a bioinformatics course in an MSc (120 ECTS), however, we recommended a teaching load of at least 8 ECTS, with anything lower than 6 CFU strongly discouraged, and with an effort to increase the time devoted to practicals to 3 ECTS. Furthermore, taking into account the results of the survey, in which we found that frequently, custom content is delivered by the teacher, in our recommendations, we identified knowledge and essential skills to be delivered indicating the number of hours of front lessons/classroom exercises suggested for each main subject, and suggesting the non-essential topics that might be replaced by others freely selected by the teachers. The recommendations issued by BITS are reported in Tables 2–5.

**Table 2:** Recommended contents for bioinformatics courses embedded in a study path belonging to a BSc degree in life science and medicine scientific area.

1) Elements of computer science and essential statistics (8 hours): (a) Elements of computer architecture, hardware, basic software, and application software (also networks and cloud) (b) Algorithms; computational power, and efficiency of algorithms (c) Elements of probability and statistics (Mean, Median, A priori and a posteriori probability, Bayes’ Theorem, Likelihood)
2) Data organization and management (8 hours): **(a) Databases (DB) and Database Management System (DBMS): (Structure of DBs, relational DBs, design and data entry, access and query methodologies)** **(b) Genomic and proteomic databases:** **(i) GenBank—ENA—DDBJ (INSDC): file entry format, sequence entry, sequence search** **(ii) Genome browsers: ENSEMBL, UCSC** **(iii) UniprotKB (Swiss-Prot, TrEMBL)** **(iv) Exercises: accessing and cross-searching sequences and data**
**3) The data analysis (20 hours):** **(a) Sequence comparison:** **(i) Alignment (local or global): definitions; optimal alignment, alignment scores, Substitution Matrices** **(ii) Alignment algorithms: dynamic programming, heuristic algorithms (BLAST, FASTA)** **(iii) Exercises: similarity searches in databases** **(b) Phylogenies and pattern prediction:** **(i) Multiple alignments** **(ii) Profile construction and pattern prediction** **(iii) Methods of constructing phylogenetic trees** **(iv) Exercises: database searches of profiles and patterns, multiple alignments**
4) Structural bioinformatics (12 hours): (a) Prediction of secondary structures: (i) Statistical-probabilistic methods (Chou and Fasman, GOR) (ii) Artificial intelligence-based methods (neural networks, HMM) (b) Prediction of tertiary structures: (i) Template-based approaches (ii) Template-free approaches (c) Protein-ligand interactions: (i) Docking (ii) Computer-assisted drug design

Course organization (6 ECTS–no less than 48 hours, with possibly 2 ECTS dedicated to classroom exercises). In bold are the contents considered as essential; the non-essential content may be replaced by custom content selected by the teacher.

**Table 3:** Recommended contents for bioinformatics courses embedded in a study path belonging to an MSc degree in the life science and medicine scientific area.

1) Introduction: rationale, growing importance of data in biology and medicine, the bioinformatics profession (2 hours)
**2) DNA sequencing, NGS, and reads: Data generation (6 hours):** **(a) Nucleic acid sequencing platforms: the 3 generations** **(b) From “physical” to “symbolic” data: software for “base calling”** **(c) Coverage, quality of reads, FASTQ, and FASTA formats** **(d) From reads to sequence: assembly algorithms** **(e) Genome annotation**
**3) Data organization and management (12 hours):** **(a) Databases (DBs) and DBMS: Structure of DBs, relational DBs, design and data entry, access and query methodologies** **(b) Genomic and proteomic databases:** **(i) Genomic databases (GenBank—ENA—DDBJ)** **(ii) Proteomic databases (UniprotKB, Swiss-Prot, TrEMBL—PDB)** **(iii) Genome browsers: ENSEMBL, UCSC** **(iv) Exercises: accessing and cross-referencing sequences and data**
**4) The analysis of data (30 hours)** **(a) Comparison of sequences:** **(i) Alignment (local or global): definitions; optimal alignment, alignment scores, Substitution Matrices** **(ii) Exact alignment algorithms: dynamic programming** **(iii) Heuristic algorithms (BLAST, FASTA), similarity searches in databases** **(b) Phylogenies and pattern prediction:** **(i) Multiple alignments** **(ii) Profile construction and pattern prediction** **(iii) Methods of constructing phylogenetic trees** **(iv) Exercises** **(c) Transcriptome analysis:** **(i) Annotation of alternative genes and transcriptomes** **(ii) Analysis of RNA-seq data** **(iii) The structure of RNA** **(iv) Exercises** **(d) Proteomics and protein interactions:** **(i) Protein identification** **(ii) Protein interaction databases, protein interaction prediction methods** **(iii) Exercises** (e) Systems biology: the role of models and nods to network biology
**5) Structural bioinformatics (14 hours):** **(a) Prediction of secondary structures** **(b) Prediction of tertiary structures** (c) Protein–ligand interactions: (i) Docking (ii) Computer-assisted drug design

**Fundamental prior knowledge:**

0.1 Elements of computer architecture, hardware, basic software, and application software (including networks and cloud).

0.2 Algorithms; computational power and efficiency of algorithms.

0.3 Elements of probability and statistics (Mean, Median, A priori and a posteriori probability, Bayes’ Theorem, Likelihood).

0.4 Elements of Machine learning and clustering.

Course organization (8 CFUs–no less than 64 hours, with possibly 3 ECTS dedicated to classroom exercises). In bold are the contents considered essential; the non-essential content may be replaced by custom content selected by the teacher.

**Table 4:** Recommended contents for bioinformatics courses embedded in a study path belonging to a BSc degree in computer science-engineering scientific area.

1) Introduction to bioinformatics: rationale, growing importance of data in biology and medicine, the bioinformatics profession (2 hours)
**2) Basic elements of molecular biology and genomics (2 hours)**
**3) DNA sequencing, NGS, reads (4 hours):** **(a) Nucleic acid sequencing platforms: the 3 generations** **(b) From “physical” to “symbolic” data: software for “base calling”** **(c) Coverage, quality of reads, FASTQ, and FASTA formats** **(d) From reads to sequence: assembly algorithms**
**4) Genomic and proteomic databases: structure and use (8 hours):** **(a) GenBank** **(b) UniprotKB (Swiss-Prot, TrEMBL)** **(c) Exercises: accessing and cross-searching sequences and data**
**5) Basics of language programming (e.g., R/Python/Julia (1 of choice) (8 hours)**
**6) Sequence alignment algorithms (8 hours)** **(a) Alignment (local, global, multiple): definitions; optimal alignment, alignment scores, Substitution Matrices** **(b) Alignment algorithms: dynamic programming, heuristic algorithms (BLAST, FASTA)** **(c) Exercises: similarity searches in databases, pairwise, and multiple alignment**
7) Algorithms for 2D and 3D structure prediction of proteins (8 hours): (a) Secondary structure prediction: Statistical-probabilistic methods (Chou and Fasman, GOR) (b) Tertiary structure prediction: Template-based and template-free approaches
8) Generation and analysis of key homology data and development of pipelines for bioinformatics (8 hours): (a) Genomics, proteomics, interactomics data (b) Hints at NGS, Microarray, Mass Spectrometry technologies (c) Hints at data analysis methodologies: Case-control experiments, Classification, Clustering (d) Main packages and workflows for bioinformatics (e.g., Bioconductor, Galaxy, Bio-Linux, Bio-Python)

Course organization (6 CFUs–no less than 48 hours, with possibly 2 ECTS dedicated to classroom exercises). In bold are the contents considered as essential; the non-essential content may be replaced by custom content selected by the teacher.

**Table 5:** Recommended contents for bioinformatics courses embedded in a study path belonging to an MSc degree in the computer science-engineering scientific area.

1) Introduction to bioinformatics (2 hours)
**2) Basic concepts of molecular biology and genomics (4 hours)**
**3) Data generation (4 hours):** **(a) Nucleic acid sequencing platforms: the 3 generations** **(b) From “physical” to “symbolic” data: sw of “base calling”** **(c) Coverage, quality of reads, FASTQ, and FASTA formats** **(d) From reads to sequence: assembly algorithms** **(e) Genome annotation**
**4) Data organization and management (6 hours):** **(a) Genomic and proteomic databases: entry file format, sequence entry, sequence search:** (i) GenBank—ENA—DDBJ (INSDC) (ii) UniprotKB (Swiss-Prot, TrEMBL)
5) Programming in R/Biopython/Julia (Choice) (8 hours)
6) Data structures for bioinformatics: Suffix and affix trees, graphs, Burrows–Wheeler transform (6 hours)
**7) The analysis of data (24 hours):** **(a) Comparison of sequences:** **(i) Alignment (local or global): definitions; optimal alignment, alignment scores, Substitution Matrices** **(ii) Exact alignment algorithms: dynamic programming** **(iii) Heuristic algorithms (BLAST, FASTA), similarity searches in databases** **(b) Phylogenies and pattern prediction:** **(i) Multiple alignments** **(ii) Profile construction and pattern prediction** **(iii) Methods of constructing phylogenetic trees** **(iv) Exercises** **(c) Transcriptome analysis:** **(i) Annotation of alternative genes and transcriptomes** **(ii) Analysis of RNA-seq data** **(iii) The structure of RNA** **(d) Proteomics and protein interactions:** **(i) Protein identification** **(ii) Protein interaction databases, protein interaction prediction methods** **(iii) Exercises** (e) Systems biology: the role of models
8) Structural bioinformatics (10 hours): (a) Prediction of secondary structures (b) Prediction of tertiary structures (c) Protein–ligand interactions: (i) Docking (ii) Computer-assisted drug design
9) Generation and analysis of key omics data and Pipeline development for bioinformatics (8 hours): (a) Genomics, proteomics, and interactomics data (b) Hints at NGS, Microarray, Mass Spectrometry technologies (c) Hints at data analysis methodologies: Case-control experiments, Classification, Clustering (d) Main packages and workflows for bioinformatics (e.g., Bioconductor, Galaxy, Bio-Linux, Bio-Python)

Course organization (8 CFUs–no less than 64 hours, with possibly 3 ECTS dedicated to classroom exercises). In bold are the contents considered as essential; the non-essential content may be replaced by custom content selected by the teacher.

### Recommendations for the teaching context for Bioinformatics courses

In order to best contextualize a bioinformatics course within a study path and facilitate the achievement of the teaching objectives, BITS believes it is helpful to accompany the list of recommended contents with the following general considerations:

1. Interdisciplinary skills are mandatory to learn bioinformatics properly. Learning an exquisitely interdisciplinary subject such as bioinformatics benefits enormously from knowledge transfer from other courses and disciplines. Such connections permit the acquisition of knowledge from other domains and a way to apply bioinformatic tools and techniques. If done correctly, interdisciplinarity will thus ensure the pertinency of formulated questions, the choice of the most appropriate tools, and the applicability of the answers.
  The choice of the disciplines to connect to the bioinformatic course will be consistently different between the BIO and INFO realms. In the first case, i.e., for bioinformatics courses delivered in study paths belonging to the life science/medicine area, BITS recommends that students strengthen their knowledge of hard science and skills belonging to the theoretical, formal, and technical spheres. These would be mathematics, statistics, computer science, and basic programming skills to understand theoretical (functions, algorithms, statistical tests) and practical (calculus, the basics of Unix/Linux, and command line) bases on which to hinge the foundations of the course. Conversely, for bioinformatics courses delivered in study paths belonging to the computer science/engineering area, BITS considers it mandatory to expose students to soft sciences and experimental thinking, with an introductory course in biochemistry and biology (again, cellular and molecular).
2. Promote the introduction of bioinformatics courses in both levels of the academic degrees. The establishment of only 1 bioinformatics course in an Italian student’s entire academic career (either only in the BSc degree or, more frequently, only in the MSc degree) forces teachers to excessively shrink the topics covered in the course. Therefore, BITS envisions that a BSc study path can include at least one 6 ECTS bioinformatics course, dedicated to the fundamentals of bioinformatics, and that the MSc study path can include a bioinformatics course of at least 8 CFU, dedicated to contemporary applications, such as “omics” sciences, structural bioinformatics, and systems biology.
3. Prefer hands-on teaching. It is essential that students acquire full awareness of the significance of bioinformatics procedures for research, analysis, and data processing; for this reason, it is recommended to avoid as much as possible a “black box” approach to the use of bioinformatics tools (in particular, those freely available online). Furthermore, virtualization techniques now allow running complex multistep pipelines in 1 terminal command, but they should be taught as advanced courses and not at the beginning, letting the students build their own small workflows from scratch, to better appreciate the complexity of workflow management. BITS also suggests that the teaching of bioinformatics should not be limited to a purely descriptive approach, but should include examples of research projects, appropriately sized to the skills to be acquired. This would enable students not only to apply bioinformatics tools, but also to critically evaluate the results, understanding the differences that different types of tools and approaches may have on the data obtained.
4. Make students aware of the importance of Open Science principles. BITS recommends raising students’ awareness of the importance of proper management, sharing, and reproducibility of all research outputs (scientific data, software, workflows, training, etc.) by introducing the principles of “data FAIRness” [9], “data sharing” [10], “open science” (https://data.consilium.europa.eu/doc/document/ST-9526-2016-INIT/en/pdf), and ethics in scientific research [11]. For this reason, BITS suggests that these and other topics (including combating racial and gender biases in science) be included in special courses, additional to the bioinformatics course, to be delivered early in the education path of the students.

## Conclusions

BITS has encouraged its members and faculty colleagues in charge of bioinformatics courses to adhere to these recommendations and to promote their adoption within the various academic teaching bodies. Moreover, we shared these recommendations with 2 Italian bodies that are involved in evaluating the quality of teaching at the academic level, both in the biological and computer science fields (CBUI: http://www.cbui.it/wp/ and GRIN: http://www.grin-informatica.it/opencms/opencms/grin). Both have positively evaluated the work done, beginning a path of collaboration with the society for future evaluation of study paths.

BITS will periodically revise these recommendations based on the evolution of the discipline, on the feedback received from the faculties, and considering the development of bioinformatics in the international context. In particular, attention will be paid to suggestions arising from associations dedicated to education in bioinformatics, for example, the training platform of ELIXIR (the European bioinformatics infrastructure supporting life sciences, in which some of us collaborate: https://elixir-europe.org/platforms/training), and GOBLET (Global Organization for Bioinformatics Learning, Education and Training: https://www.mygoblet.org/), of which BITS is a member, which hosted in October 2021, as part of the joint GOBLET & EMBnet Annual General Meeting 2021, the presentation of these data, and that has issued materials to support the teaching of bioinformatics [12,13]. We will also be happy to align with other guidelines and suggestions issued by other official entities that can improve the quality of bioinformatics education in Italy. We hope that sharing these recommendations here will enable us to gather international viewpoints that will help us in further improving these recommendations for the future. Additionally, we hope that the feedbacks provided by our associates and the faculties, and the guidelines issued by national/international associations dedicated to education in bioinformatics will assist us in planning improvements of these recommendations in the future. Our intent is precisely to monitor over time whether and how these recommendations will be taken into consideration by the Italian bioinformatics community and to intervene later to include correctives that will possibly improve their effectiveness.

## Supporting information

S1 Data Raw data of the survey. The output of the Google form is made available in anonymized format (Italian only).
(XLSX)

S1 Text Glossary. Explanation of the terminology used in this contribution and of the meaning of some terminology referred to academic classifications currently used in Italy.
(DOCX)

S1 Fig Details of the participants to the survey. Panel (a): Detailed list of the Universities that participated to the survey, with information concerning their geographical location and the number of the answers collected for each University. Panel (b): Visual localization of universities on the map of Italy (map source: https://commons.wikimedia.org/wiki/File:Map_of_Italy-it.svg).
(TIF)

S2 Fig **Detailed distribution of the teachers (panel (a)) and of the course (panel (b)) with respect to the Italian academic disciplines classification (SSD).** For the meaning of the codes, please refer to: https://www.cun.it/uploads/storico/settori_scientifico_disciplinari_english.pdf.
(TIF)

S3 Fig Frequency of the topics treated in the bioinformatics courses. Since multiple answers were allowed in the survey, the total is higher than 100%.
(TIF)
