# Supplementary material for: An overview of bioinformatics courses delivered at the academic level in Italy: Reflections and recommendations from BITS
Source: PLoS Comput Biol. 2023 Feb 13;19(2):e1010846. doi: 10.1371/journal.pcbi.1010846 (PMC9924992; doi:10.1371/journal.pcbi.1010846)
Supplement: S1 Text — Explanation of the terminology used in this contribution and of the meaning of some terminology referred to academic classifications currently used in Italy. (DOCX) [file pcbi.1010846.s002.docx]

To avoid ambiguities and misunderstandings, we would like to clarify the meaning of the terminology used in the current contribution.

● The term "course" means "a series of classes on a particular subject (in our case, bioinformatics), usually lasting a whole semester".

● The term "study path" means "the set of courses that a student must follow to obtain an academic degree in a particular field".

● The term "bachelor's degree" (BSc) means "the first academic degree that an undergraduate student can achieve at the academic level". In Italy, students must have a high school diploma to enter this degree level. The regular duration of this degree is three years.

● The term "master's degree" (MSc) means "the second academic degree that an undergraduate student can achieve at the academic level". In Italy, students must have a bachelor's degree to enter this level. The regular duration of this degree is two years.

In Italy, the disciplinary affiliation, i.e., the scientific placement that each lecturer or course assumes in the university system, is divided into disciplinary groupings. According to Article 15 of Law 240 of 2010, the structure of these groupings is divided into three levels of increasing specificity names:

● Macro Sectors (MSC = 86);

● Sectors (SC = 190);

● Scientific Disciplinary Sectors (SSD = 383).

The Macro Sectors refer to 14 CUN (Consiglio Universitario Nazionale – Italian National University Council) scientific areas.

SSD are essential because they are the basis of many organizational aspects of universities. For example, they give a way to properly articulate courses (see our previous definition) and define the goals to obtain a degree in a particular discipline. To this extent, each study path in a given discipline must contain a certain amount of ECTS attributed to courses marked by the SSD selected as most relevant to that discipline. (Partial translation of the text found on the official Web site of the Italian Ministry of the Instruction, University and Research (MIUR) (Italian only): <https://www.miur.gov.it/settori-concorsuali-e-settori-scientifico-disciplinari>)

Each SSD is characterized by an abstract summarizing the scientific topics of that SSD. Bioinformatics appears only as a keyword within the abstracts of the BIO/10, BIO/11, and ING-INF/06 SSDs (those being Biochemistry, Molecular Biology, and Electronic and Computer Bioengineering). Interestingly, bioinformatics courses currently present in Italy are often associated with those three SSDs plus two others: Computer Science (INF/01) and computer engineering (ING-INF/05).
