# Supplementary figures and images for: An overview of bioinformatics courses delivered at the academic level in Italy: Reflections and recommendations from BITS
Source: PLoS Comput Biol. 2023 Feb 13;19(2):e1010846. doi: 10.1371/journal.pcbi.1010846 (PMC9924992; doi:10.1371/journal.pcbi.1010846)

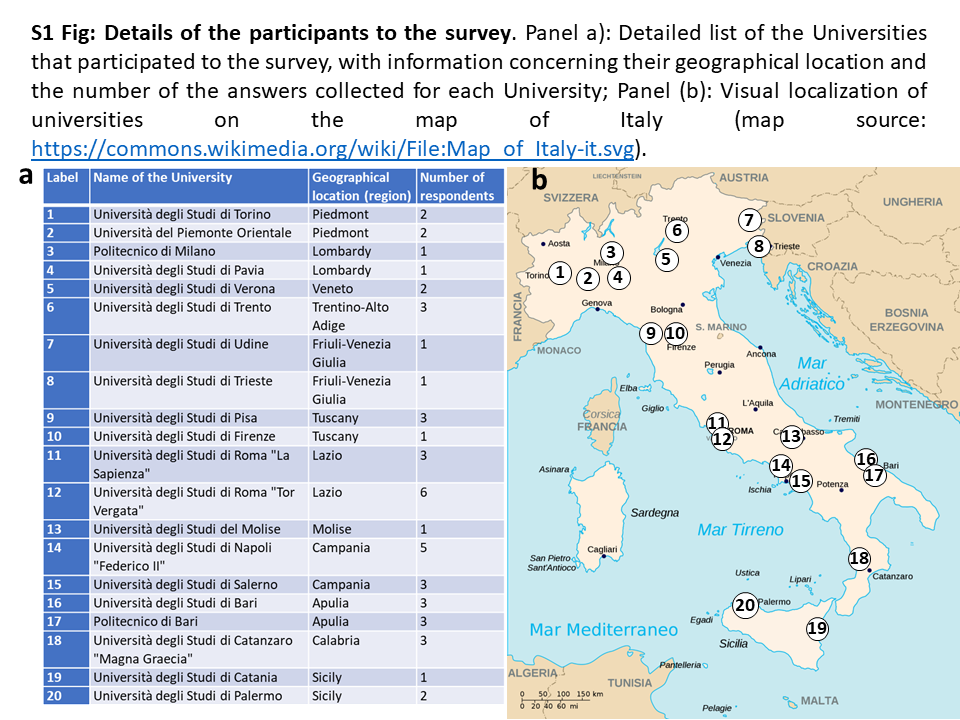

Supplement: S1 Fig — Panel (a): Detailed list of the Universities that participated to the survey, with information concerning their geographical location and the number of the answers collected for each University. Panel (b): Visual localization of universities on the map of Italy (map source: https://commons.wikimedia.org/wiki/File:Map_of_Italy-it.svg). (TIF) [file pcbi.1010846.s003.tif]

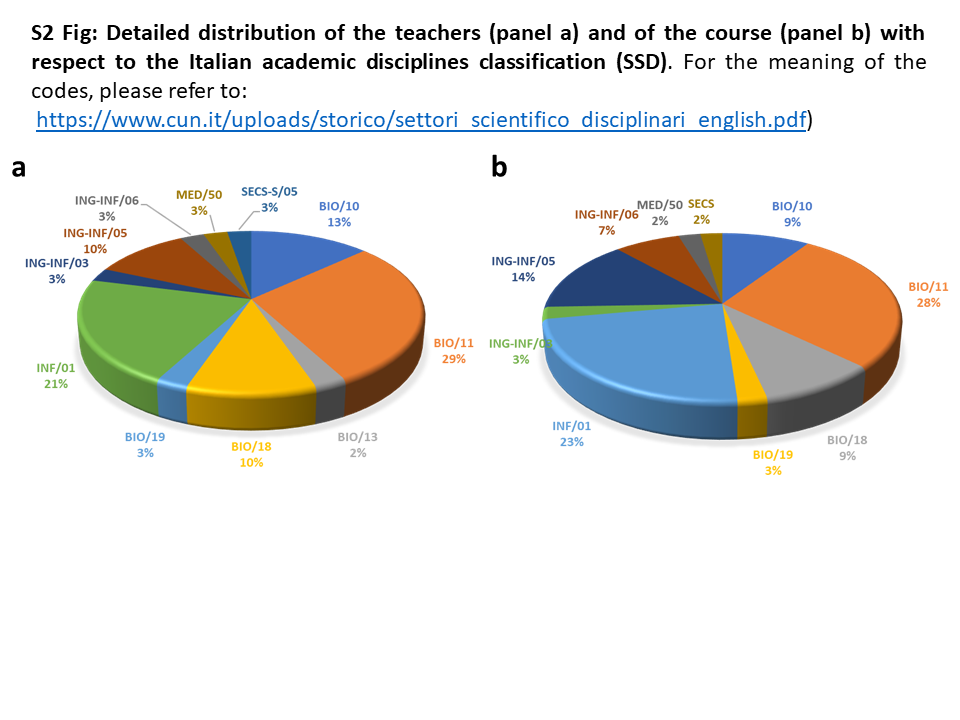

Supplement: S2 Fig — Detailed distribution of the teachers (panel (a)) and of the course (panel (b)) with respect to the Italian academic disciplines classification (SSD). For the meaning of the codes, please refer to: https://www.cun.it/uploads/storico/settori_scientifico_disciplinari_english.pdf. (TIF) [file pcbi.1010846.s004.tif]

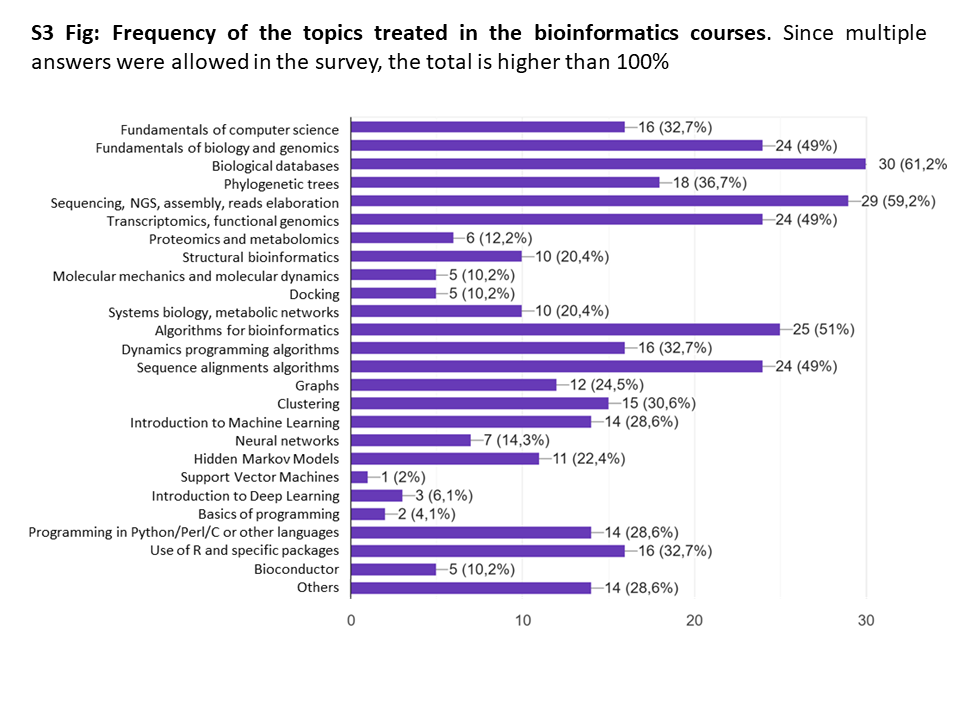

Supplement: S3 Fig — Since multiple answers were allowed in the survey, the total is higher than 100%. (TIF) [file pcbi.1010846.s005.tif]
